# Analysis of Influencing Factors of Prehypertension and Its Development in Occupational Population

**DOI:** 10.3389/ijph.2025.1608206

**Published:** 2025-03-14

**Authors:** Wenyi Zhao, Lili Du, Wanyue Qiu, Gang Wang, Dinglun Zhou

**Affiliations:** ^1^ West China School of Public Health, The Fourth Hospital of West China, Sichuan University, Chengdu, China; ^2^ Sichuan Center for Disease Control and Prevention, Chengdu, China; ^3^ Biomaterials Engineering Research Center, Sichuan University, Chengdu, China

**Keywords:** occupational population, prehypertension, hypertension, BMI, health promotion

## Abstract

**Objective:**

To determine the prevalence of prehypertension in the occupational population and the risk factors associated with the progression of hypertension.

**Methods:**

Data were collected from 13,791 people who attended occupational health examinations in Chengdu, Deyang and Mianyang in 2019 and 2023. Descriptive statistics were used to analyze prevalence and progression rates, and logistic regression was applied to assess factors influencing the progression from prehypertension to hypertension.

**Results:**

The prevalence of prehypertension was 53.66% in 2019 and 55.46% in 2023. Data from 2023 indicated that 14.05% of individuals with prehypertension had developed hypertension. Chi-square analysis revealed statistically significant associations between prehypertension progression and factors such as gender, age, BMI, enterprise size and exposure to occupational harmful factors. Logistic regression identified male gender, older age, higher BMI, and smaller enterprise size as significant risk factors for hypertension progression among individuals with prehypertension.

**Conclusion:**

The prevalence of prehypertension is high among occupational populations in China, with higher susceptibility noted among men and individuals with elevated BMI. Occupational health intervention strategies should be developed to manage and prevent the progression of hypertension.

## Introduction

Hypertension is a major risk factor for a wide range of chronic non-communicable diseases (NCDs), particularly cardiovascular diseases, and presents a significant global public health burden. In China, approximately 27.5% of adults, or roughly 300 million people, are living with hypertension, with this number still on the rise [1]. Of these individuals, about 70% are part of the working population [2], highlighting a strong link between workforce health and the nation’s socioeconomic stability. As the world’s largest labor force, China had over 750 million employed individuals in 2021, representing 53.2% of its total population [3]. These statistics underscore the critical importance of managing hypertension within the occupational sector to promote both public health and sustainable development.

Prehypertension, which serves as an intermediate stage between normal blood pressure and clinical hypertension, is an independent risk factor for both hypertension and cardiovascular diseases. Studies have shown that the risk of individuals with prehypertension developing high blood pressure within 4 years is as high as 52% [4]. A 2018 study by the National Center for Cardiovascular Diseases found that the prevalence of prehypertension among residents aged 18 and older was as high as 41.3% between 2012 and 2015, with the number of individuals at risk continuing to rise [5]. The main risk factors include smoking, alcohol consumption, poor diet, lack of exercise, and psychological stress [6]. Indicators such as body mass index (BMI) [7], fasting blood glucose (FPG), total cholesterol (TC), triglycerides (TG), high-density lipoprotein cholesterol (HDL-C), and low-density lipoprotein cholesterol (LDL-C) are independent risk factors for cardiovascular diseases in people with prehypertension. Understanding the harm caused by abnormal blood pressure is essential for early intervention, which can improve the health of individuals with prehypertension and effectively prevent its progression to full hypertension [8]. Early intervention is crucial, aligning with contemporary medical approaches aimed at reducing cardiovascular morbidity. Thus, strategies to prevent the onset of prehypertension and to control its progression to hypertension are vital for mitigating hypertension-related health burdens.

This study utilizes occupational health examination data from 2019 to 2023, collected from populations in Chengdu, Deyang, and Mianyang, to analyze the prevalence of hypertension, monitor changes over 4 years, and identify key risk factors. The findings aim to inform scientifically driven strategies for workplace health promotion and support targeted health management interventions within occupational settings.

## Methods

### Study Subjects

Enterprises regularly conduct health examination for employees, primarily to detect occupational diseases or symptoms at an early stage. This examination is typically performed once a year. The items and contents of the physical examination are determined based on the occupational hazard factors, potential risks, and clinical manifestations relevant to the occupational environment. In addition to these, routine medical examinations are also offered to employees, usually as part of their benefits package. The medical examination institutions record occupational information, including the type of work, harmful factors exposured in occupational environment, and the size of the enterprise. However, exposure doses to occupational hazards are not provided, and there is a lack of detailed behavioral data collection.

A dedicated information management system is used to collect occupational health examination data from various physical examination institutions, enabling the establishment of electronic health records for employees. This system became operational in 2018, and by 2019, many companies began uploading medical results to the system. The health examination data for Chengdu, Deyang, and Mianyang were exported from this information system. Using a unique ID number for tracking purposes, individuals who underwent health examination in both 2019 and 2023 were included in the study. The target population consisted of manufacturing workers, with the primary occupational hazard factors being physical factors, dust, and chemical exposures.

A paired design was used to analyze individuals who were identified as prehypertensive in 2019 and progressed to hypertension by 2023, with the aim of exploring related influencing factors. Before blood pressure measurement, participants were instructed to relax and rest in a quiet environment for at least 5 minutes. Blood pressure was measured in a seated position using the upper arm cuff method, ensuring the cuff was at heart level, and both arms were evaluated. The highest blood pressure reading was recorded. Hypertension classification followed the 2024 Chinese Hypertension Prevention and Control Guidelines [9], categorizing participants as normotensive, prehypertensive, or hypertensive.

Participants’ height and weight were measured, and their Body Mass Index (BMI) was calculated. BMI was classified into the following categories: underweight (<18.5), normal (18.5–23.99), overweight [10–14], and obese (≥28) [15].

### Statistical Analysis

Data were processed using R 4.1.0 for querying, duplicate checks, and correction of logical errors. IBM SPSS 22.0 software was used to analyze the factors influencing the progression from prehypertension in 2019 to hypertension in 2023 among the occupational population. Rate comparisons were conducted using the chi-square (χ^2^) test, while multivariate analysis was performed using unconditional logistic regression. Statistical significance was defined as *P* < 0.05. Missing data were handled according to the actual sample size.

## Results

### General Demographic Characteristics

A total of 13,791 individuals underwent health examinations, including 11,175 men (81.03%) and 2,616 women (18.97%). The participants’ ages ranged from 18 to 70 years, with 4,227 individuals under 40 years, 4,926 between 40 and 49 years, 4,385 between 50 and 59 years, and 253 aged 60 and above. Regarding Body Mass Index (BMI), 364 individuals were classified as underweight, 6,338 as normal weight, 5,438 as overweight, and 1,651 as obese. Work experience ranged from 0 to 30 years, with 7,258 individuals having less than 10 years, 4,317 with 10–19 years, 1,470 with 20–29 years, and 690 with 30 or more years. Companies were categorized by size as micro (1,248 individuals), small (6,558), medium (3,436), and large (2,549). Among the participants, 9,942 cases were exposed to a single occupational hazard factor, and 3,849 cases were exposed to multiple occupational hazard factors (Table 1).

**TABLE 1:** General demographic characteristics of the occupational population (China, 2023).

Demographic characteristics	n	Constituent ratio (%)
Age (year)		
<40	4,227	30.65
40∼49	4,926	35.72
50∼59	4,385	31.80
≥60	253	1.83
Gender		
Male	11,175	81.03
Female	2,616	18.97
BMI		
Underweight	364	2.64
Normal	6,338	45.96
Overweight	5,438	39.43
Obese	1,651	11.97
Years of Service		
<10	7,258	52.84
10∼19	4,317	31.43
20∼29	1,470	10.71
≥30	690	5.02
Enterprise Size		
Micro	1,248	9.05
Small	6,558	47.55
Medium	3,436	24.91
Large	2,549	18.49
Exposure to occupational harmful factors		
Single harmful factor	9,942	72.09%
Multiple harmful factor	3,849	27.91%

### Blood Pressure Composition in 2019 and 2023

In 2019, the proportion of individuals with prehypertension was 53.66%, while those with hypertension accounted for 15.57%. Data from 2023 show that the proportion of prehypertension slightly increased to 55.46%, with hypertension at 15.79%. A chi-square (c^2^) test was performed to compare the blood pressure composition between 2019 and 2023 (see Table 2). Although statistically significant, the actual difference in values is minor, suggesting that the change over time is not substantial.

**TABLE 2:** Blood pressure composition in 2019 and 2023 (China, 2019 and 2023).

Year	Normal	Prehypertension	Hypertension	χ^2^	P value
2019	30.77	53.66	15.57	13.792	<0.001
2023	28.75	53.46	15.79		

### Proportion of Prehypertension Progressing to Hypertension by Demographic Characteristics in Occupational Populations

Based on blood pressure data from two occupational health screenings conducted in 2019 and 2023, we analyzed changes in blood pressure among the same employees over time. Among workers identified as prehypertensive in 2019 (n = 7,400), 1,040 individuals progressed to hypertension by 2023, resulting in a progression rate of 14.05%. We further compared the rates of prehypertension progressing to hypertension across various demographic groups, including age, gender, BMI, company size, exposure to occupational harmful factors. The differences in progression rates were statistically significant across all categories (*P* < 0.001). See Table 3 for details.

**TABLE 3:** Proportion of prehypertension progressing to hypertension by demographic characteristics in occupational Populations (China, 2023).

Variable	Sample size	Progression (%)	χ^2^	P Value
Age (year)^a^			118.489	<0.001^a^
<40	2,221	7.70		
40∼49	2,563	14.98		
50∼59	2,469	18.19		
≥60	147	24.49		
Gender			16.427	<0.001
Male	6,176	14.78		
Female	1,224	10.38		
BMI^a^			115.900	<0.001^a^
Underweight	154	7.79		
Normal	3,225	10.45		
Overweight	3,069	14.76		
Obese	952	24.47		
Enterprise Size			18.259	<0.001^a^
Micro	678	15.78		
Small	3,635	13.26		
Medium	1750	12.57		
Large	1,337	17.28		
Exposure to occupational harmful factors			4.687	0.030
Single harmful factor	5,316	13.51		
Multiple harmful factor	2084	15.45		

^a^ Indicates trend χ2.

### Multivariate Analysis of Prehypertension Progression to Hypertension

A multivariate logistic regression analysis was conducted with gender, age, BMI, company size, exposure to occupational harmful factors as independent variables, and the progression of prehypertension to hypertension among the occupational population as the dependent variable. The results indicated that male gender (OR = 1.442), age (OR = 1.051), BMI (OR = 1.112), and working in a small enterprise (OR = 1.427) are all risk factors for hypertension among the occupational population. See Table 4.

**TABLE 4:** Analysis of factors influencing the progression from prehypertension to hypertension in occupational Populations (China, 2023).

Variable	B	S.E.	Wald	Sig	0R	Exp (B) lower limit	Exp (B) upper limit
Male	0.366	0.103	12.763	<0.001	1.442	1.180	1.764
Age	0.050	0.004	137.217	<0.001	1.051	1.043	1.060
BMI	0.106	0.011	96.531	<0.001	1.112	1.089	1.136
Enterprise Size			20.409	<0.001			
Enterprise Size (small)	0.355	0.105	11.545	0.01	1.427	1.162	1.752
Enterprise Size (medium)	0.195	0.131	2.212	0.137	1.215	0.940	1.571
Enterprise Size (large)	−0.025	0.089	0.078	0.780	0.975	0.819	1.162
Exposure to occupational harmful factors (Multiple)	−0.139	0.075	3.449	0.063	0.870	0.752	1.008
Constant	−7.044	0.371	360.561	<0.001	0.001		

## Discussion

The occupational population represents a significant demographic in China, with their health closely tied to socioeconomic stability. Elevated blood pressure in this group is influenced not only by societal and lifestyle factors, such as increasing life pressures and irregular dietary habits, but also by occupational characteristics like prolonged sedentary work, workplace stress, and job intensity. Prehypertension, initially identified by the Joint National Committee on Prevention, Detection, Evaluation, and Treatment of High Blood Pressure (JNC 7) [16] in the United States, has gained increasing recognition in China. As prehypertension rates rise among Chinese adults, understanding the factors driving its progression is critical for both clinical practice and public health. Research has shown that deaths related to hypertension surpass those linked to other modifiable risk factors, and the risk of cardiovascular disease increases in a logarithmic-linear fashion with incremental rises in both systolic and diastolic blood pressure [17]. A 20 mmHg increase in systolic or a 10 mmHg rise in diastolic blood pressure doubles the risk of stroke, heart disease, or other cardiovascular conditions [18].

This study utilizes health examination data from occupational cohorts across Chengdu, Deyang, and Mianyang in Sichuan Province from 2019 to 2023 to assess prehypertension prevalence and progression within the working population. It identifies risk factors for progression from prehypertension to hypertension, offering insights into workplace health promotion strategies to improve health outcomes for this demographic.

The data reveal an overall prehypertension prevalence of 53.66% in 2019 and 55.46% in 2023, with only a slight increase of 1.8% over 5 years. This minor rise may be attributed to factors such as the use of antihypertensive medications, measurement methods, and staff standardization in measurement. Multivariate analysis identified gender, age, body mass index (BMI), work tenure, and company size as significant risk factors for hypertension progression. These findings align with the results of Yin R et al. [19], showing that men exhibit a higher progression rate (14.78%) compared to women (10.36%). Blood pressure is a sexually dimorphic trait, and the prevalence of this condition can vary significantly between males and females throughout the lifespan [20]. This difference is due to a combination of biological (sex) and psychosocial (gender) factors [21], with estrogen in women playing a protective role in blood pressure regulation via the renin-angiotensin-aldosterone system, sympathetic nervous system, oxidative stress, and endothelial function. Furthermore, men are more likely to engage in riskier behaviors, such as smoking and drinking, which may contribute to higher blood pressure compared to women [22]. Our findings are consistent with studies by Malik KS et al. [23], showing that the prevalence of prehypertension increases with age, making older individuals more vulnerable to progression.

BMI is another critical risk factor, reflecting its strong association with adverse health outcomes in the occupational population [24]. Sedentary work, common in mentally intensive occupations, leads to BMI increases, while higher energy demands in physically intensive jobs often result in greater calorie and fat intake [14], further contributing to elevated BMI. The global prevalence of obesity increased from 3.2% to 10.8% in men and from 6.4% to 14.9% in women between 1975 and 2014. By 2025, it is projected that 18% of men and 21% of women worldwide will be obese [25]. Occupational populations, a significant portion of adults, are especially affected by overweight and obesity, both of which are linked to chronic conditions such as hypertension, dyslipidemia, diabetes, and fatty liver disease. These issues are exacerbated by work stress, sedentary lifestyles, and irregular eating habits [26]. Our results show that higher BMI correlates with an increased likelihood of transitioning from prehypertension to hypertension, emphasizing the need for targeted health education, a patient-centered approach to obesity care [27], lifestyle changes, weight loss goals [14], weight management initiatives, and green dietary habits in occupational settings.

The study also highlights a significant link between company size and hypertension progression, with prehypertensive employees in smaller companies more likely to develop hypertension. This suggests discrepancies in health management between companies of different sizes, underscoring the need for smaller enterprises to implement comprehensive health promotion initiatives and occupational health policies. As noted by Micheli GJL et al. [10], the relationship between workers’ health, safety, and welfare, and the role of social dialogue in defining workers’ health and safety experiences, is often overlooked in occupational safety and health (OSH) research, especially in small and medium-sized enterprises (SMEs). Improving safety conditions in SMEs is crucial, requiring resources to be allocated effectively to OSH [11, 12]. Smaller enterprises often provide only basic legal benefits, lack professional occupational health management systems and personnel, and are less able to identify and mitigate occupational health risks. This exposes employees to various health hazards, with fewer opportunities for early detection of prehypertension. In contrast, large enterprises may have higher work intensity and pressure, and more complex workflows, leading to long-term stress exposure, which can also raise blood pressure [28]. Therefore, regardless of company size, there are factors that influence employee blood pressure, reinforcing the need for enterprises to enhance health management systems tailored to their size to ensure employee health.

This study has limitations. First, baseline data lacked information on certain behavioral factors, and the use of antihypertensive medications was not recorded. Lifestyle factors such as smoking, alcohol consumption, excessive salt intake, and lack of physical activity are all known risk factors for prehypertension [6]. Additionally, the data analyzed only cover 2019 and 2023, limiting the ability to track dynamic changes over time. Future research could implement pilot studies at selected health examination institutions or companies, enhancing health data collection and including behavioral surveys for more comprehensive analysis. Follow-up surveys on prehypertension progression should also consider occupation-specific factors such as job stress [29], shift work, and job type, for a more thorough understanding of hypertension risk factors in the occupational population. Furthermore, research indicates that individuals with hypertension or prehypertension should avoid environments exposed to harmful substances, such as disulfides, lead, cadmium, arsenic, and mercury. Workers in industries like high-voltage lines and coal mines are at higher risk of abnormal blood pressure [30].

In conclusion, prehypertension remains prevalent in occupational populations, with progression influenced by factors such as age, gender, BMI, work tenure, company size, and exposure to occupational hazards. Men and individuals with higher BMI are key targets for workplace health promotion. Early intervention to prevent blood pressure elevation in prehypertensive individuals is essential to improve health outcomes within this population. Our findings provide valuable guidance for workplace health promotion efforts, potentially improving health within occupational groups.
